# Editorial: Novel insights into liver injury: mechanisms, pathophysiology, and therapeutic strategies

**DOI:** 10.3389/fmed.2025.1542598

**Published:** 2025-02-18

**Authors:** Zibing Qian, Junfeng Li

**Affiliations:** ^1^The First Clinical Medical College of Lanzhou University, Lanzhou, China; ^2^Institute of Infectious Diseases, The First Hospital of Lanzhou University, Lanzhou, China; ^3^Department of Hepatology, The First Hospital of Lanzhou University, Lanzhou, China

**Keywords:** liver disease, immunoregulation, biomarker, drug, treatment

Liver disease remains a major global health burden, with high morbidity and mortality rates (1). In recent years, continuous progress in biomedical fields such as immune regulation, biomarkers, and new drug research and development has brought new opportunities for the diagnosis and treatment of liver diseases. The complex interactions between diverse immune cell populations in the liver work together to maintain its immune function, ensuring the effective clearance of various infections and abnormal cells (2). Furthermore, the search for specific biomarkers has great significance for the diagnosis, prognosis assessment, and treatment monitoring of liver diseases. Simultaneously, drug development has also brought new hope for the treatment of liver diseases. In-depth study of liver immunity, potential biomarkers, and new drug development is helpful to improve our understanding of the pathogenesis of liver diseases and provide new targets and methods for disease prevention, diagnosis, and individualized treatment.

Notably, our Research Topic explores emerging liver disease regulatory mechanisms, potential biomarkers, and therapeutic strategies, providing a roadmap for future liver disease research and clinical translation. Ajith et al. proposed that previous research focuses on the use of immune cells to prevent autoimmune diseases while using specific blockers to inhibit their activity or alter their phenotypes to reduce the immunosuppressive properties in the tumor microenvironment. However, in recent years, research into the mechanism of programmed death of immune cells has provided a new idea for the treatment of immune diseases (3). It has been found that there are various ways that programmed death of immune cells occurs, such as apoptosis, necroptosis, autophagy, pyroptosis, and ferroptosis. Proper regulation helps to maintain immune homeostasis, but the imbalance may also lead to autoimmune diseases. Immune cells can limit their attack effects on target organs through specific programmed death mechanisms, such as autophagy and necroptosis, thus avoiding tissue damage (4, 5). Secondly, excessive programmed death of immune cells may weaken the body's ability to fight infection and increase susceptibility to infection (6). This indirectly suggests that the precise regulation of the immune cell programmed cell death pathway can inhibit the attack of immune cells on the liver to a certain extent, thus protecting liver cells from further damage. It has become clear that targeted modulation of the programmed death pathway of immune cells is a new frontier of immunotherapy research. By enhancing the survival and function of immune cells, it promotes the clearance of abnormal cells while reducing damage to normal tissues (7). This strategy is applicable to cancer, autoimmune diseases and chronic infections, demonstrating good adaptability and versatility. Notably, targeted modulation of the immune cell programmed cell death pathway may become a new strategy for the treatment of immune-mediated liver injury. Compared with traditional immune modulation, this approach can regulate the immune system more selectively and reduce the occurrence of adverse effects.

The regulation of immune cells affects the progression and prognosis of almost all liver diseases, and exploring this mechanism is crucial for biomarker development. The discovery of biomarkers not only reflects the status of immune cells but also provides an important basis for the diagnosis and monitoring of diseases. The occurrence of diseases often triggers corresponding biological changes, which can be reflected by the detection of specific biomarkers. Currently, traditional liver function indicators such as alanine transaminase (ALT) and aspartate transaminase (AST) are still commonly used in the clinic for the diagnosis and monitoring of liver diseases. However, such indicators lack specificity and have limited diagnostic ability, particularly for early liver injury. Our Research Topic includes two original articles that explore novel biomarkers for liver disease. These emerging biomarkers have the potential to improve the diagnosis, prognosis assessment, and treatment monitoring of liver diseases compared to traditional markers. Felgendreff et al. compared different brain injury parameters in plasma and tissues with the progression of acute liver failure (ALF). The ALF model showed a significant increase in plasma glial fibrillary acidic protein (GFAP) levels after 24 h of induction, which suggests that blood GFAP can serve as an ALF related brain injury biomarker. Song et al. noted that adrenoceptor beta 2 (ADRB2) is a promising biomarker with potential diagnostic and prognostic value in clinical cohort data. ADRB2 may alleviate alcoholic liver disease by activating the SIRT1/PPARα/PGC-1α pathway. Fibroblast growth factor 21 (FGF21) is a stress-inducing hormone that plays a beneficial role in adaptive responses to various physiological or pathological stressors (8). Sodium butyrate (NaB), a short-chain fatty acid, has received increasing attention due to its tremendous beneficial effects such as modulating antioxidant and anti-inflammatory properties (9). Yang J. et al. showed that NaB could attenuate BDL-induced liver injury and fibrosis in an FGF21-dependent manner. Emerging biomarkers are playing an increasingly important role in the diagnosis and monitoring of diseases, and we can expect these biomarkers to be used in the clinic as soon as possible.

The development of emerging marker assays provides a multifaceted boost to new drug discovery by improving diagnostic accuracy, monitoring drug response, accelerating individualized therapy, and discovering new therapeutic targets. With the rapid development of new drug research and development, a new generation of biologics is also appearing unceasingly. However, “new use of old drugs” is also a new track in drug development. Farnesoid X receptor (FXR) agonists are among the most promising NAFLD drugs, and obeticholic acid (OCA) is an FXR agonist (10). Yang Y. et al. effectively reduced high-fat diet-induced hepatic steatosis by combining an Acyl-CoA oxidase 1 (ACOX1)-specific inhibitor with low-dose obeticholic acid. The antiepileptic drug valproic acid (VPA) is effective and well tolerated in clinical use, but prolonged VPA therapy may lead to liver injury (11). Zhu et al. found that patients chronically treated with VPA exhibit metabolic abnormalities in single carbon metabolism (OCM), leading to alterations in OCM-related nutrients and an increased risk of hepatic dysfunction. This provides an important theoretical basis for preventing and guiding the clinical treatment of VPA-induced liver dysfunction. In short, based on the existing drug research foundation, the development of chemical derivatives and new dosage forms is also an effective approach to explore new uses for old drugs. This “secondary innovation” can not only save research and development costs but also make full use of previous research results to achieve the circular development of drugs.

In summary, rapid progress in biomedical fields, such as immune function regulation, biomarkers, and new drug development, has injected new impetus into the precise diagnosis and personalized treatment of liver diseases. Going forward, we will vigorously promote the sustained development of the pharmaceutical industry by attaching great importance to the cross-fertilization of disciplines, continuously exploring new biomarkers and therapeutic targets as the mainstream direction. In addition, high-tech technologies such as single-cell technology, proteomics, and metabolomics, as the most promising means of life sciences at present, have demonstrated great development potential and momentum. Single-cell sequencing can accurately detect the heterogeneity and functional changes of immune cells, helping to identify specific subpopulations and their dynamic changes (12). Proteomics utilizes mass spectrometry and bioinformatics to analyze protein expression and interactions in immune cells, providing higher-level evidence for biomarker identification and exploration of immunoregulatory mechanisms (13). The metabolic characterization of immune cells through metabolomics reveals how metabolic pathways affect immune cell function and helps to understand immune responses and related diseases. Currently, the application and research of metabolomics in the diagnosis of hereditary metabolic defects, tumors, liver diseases, cardiovascular diseases, psychiatric disorders and other diseases has made rapid development (14). In the future, these technologies will further advance our understanding of the complexity and dynamics of the immune system, provide higher-level support for the continuous advancement of biomedical fields such as biomarkers and new drug discovery, and facilitate their earlier entry into clinical applications.

## References

[1] Devarbhavi H Asrani SK Arab JP Nartey YA Pose E Kamath PS. Global burden of liver disease: 2023 update. J Hepatol. (2023) 79:516–37. 10.1016/j.jhep.2023.03.017 36990226

[2] Lohse AW Weiler-Normann C Tiegs G. Immune-mediated liver injury. J Hepatol. (2010) 52:136–44. 10.1016/j.jhep.2009.10.016 19913936

[3] Kroemer G Galassi C Zitvogel L Galluzzi L. Immunogenic cell stress and death. Nat Immunol. (2022) 23:487–500. 10.1038/s41590-022-01132-2 35145297

[4] Li X Zhang Y Wang J Li Y Wang Y Shi F. zVAD alleviates experimental autoimmune hepatitis in mice by increasing the sensitivity of macrophage to TNFR1-dependent necroptosis. J Autoimmun. (2022) 133:102904. 10.1016/j.jaut.2022.102904 36108506

[5] Lodder J Denaës T Chobert MN Wan J El-Benna J Pawlotsky JM. Macrophage autophagy protects against liver fibrosis in mice. Autophagy. (2015) 11:1280–92. 10.1080/15548627.2015.1058473 26061908 PMC4590651

[6] Linkermann A Stockwell BR Krautwald S Anders HJ. Regulated cell death and inflammation: an auto-amplification loop causes organ failure. Nat Rev Immunol. (2014) 14:759–67. 10.1038/nri3743 25324125

[7] Li J Huang Q Lv M Ma W Sun J Zhong X. Role of liensinine in sensitivity of activated macrophages to ferroptosis and in acute liver injury. Cell Death Discov. (2023) 9:189. 10.1038/s41420-023-01481-3 37353487 PMC10290152

[8] Tan H Yue T Chen Z Wu W Xu S Weng J. Targeting FGF21 in cardiovascular and metabolic diseases: from mechanism to medicine. Int J Biol Sci. (2023) 19:66–88. 10.7150/ijbs.73936 36594101 PMC9760446

[9] Li Y Liu A Chen K Li L Zhang X Zou F. Sodium butyrate alleviates lead-induced neuroinflammation and improves cognitive and memory impairment through the ACSS2/H3K9ac/BDNF pathway. Environ Int. (2024) 184:108479. 10.1016/j.envint.2024.108479 38340407

[10] Markham A Keam SJ. Obeticholic acid: first global approval. Drugs. (2016) 76:1221–6. 10.1007/s40265-016-0616-x 27406083

[11] Song W Yan X Zhai Y Ren J Wu T Guo H. Probiotics attenuate valproate-induced liver steatosis and oxidative stress in mice. PLoS ONE. (2023) 18:e0294363. 10.1371/journal.pone.0294363 37971986 PMC10653412

[12] van Galen P Hovestadt V Wadsworth Ii MH Hughes TK Griffin GK Battaglia S. Single-Cell RNA-Seq reveals AML hierarchies relevant to disease progression and immunity. Cell. (2019) 176:1265–1281.e24. 10.1016/j.cell.2019.01.031 30827681 PMC6515904

[13] Leuschner G Semenova A Mayr CH Kapellos TS Ansari M Seeliger B. Mass spectrometry-based autoimmune profiling reveals predictive autoantigens in idiopathic pulmonary fibrosis. iScience. (2023) 26:108345. 10.1016/j.isci.2023.108345 38026226 PMC10661358

[14] Nicholson JK Wilson ID. Opinion: understanding ‘global' systems biology: metabonomics and the continuum of metabolism. Nat Rev Drug Discov. (2003) 2:668–76. 10.1038/nrd1157 12904817

